# An *Epichloë* fungal endophyte regulates grain size in barley

**DOI:** 10.3389/fpls.2026.1917832

**Published:** 2026-09-10

**Authors:** Zhengfeng Wang, Wanyou Liu, Zhenjiang Chen, Feng Zhao, Yonghong Qi, Chunjie Li

**Affiliations:** 1 Institute of Plant Protection, Gansu Academy of Agricultural Sciences, Lanzhou, China; 2 Centre for Grassland Research, National Forestry and Grassland Administration, Chinese Academy of Forestry, Beijing, China; 3 State Key Laboratory of Herbage Improvement and Grassland Agro-Ecosystems; Key Laboratory of Grassland Livestock Industry Innovation, Ministry of Agriculture and Rural Affairs Engineering Research Center of Grassland Industry, Ministry of Education Gansu Tech Innovation Centre of Western China Grassland Industry Center for Grassland Microbiome, College of Pastoral Agriculture Science and Technology, Lanzhou University, Lanzhou, China; 4 Institute of Economic Crop and Malt Barley, Gansu Academy of Agricultural Science, Lanzhou, China

**Keywords:** barley, *Epichloë* endophyte, hormone signaling, metabolomics, transcriptomics

## Abstract

Grain size represents a key agronomic trait and a major determinant of barley grain yield potential. We observed that the grain size of *Epichloë bromicola*-inoculated barley plants were significantly larger than those of the uninoculated barley plants. However, the mechanisms and functions by which the endophyte fungus *E. bromicola* modulates barley grain size have not been investigated. In this study, we compared grain size between uninoculated (KE-) and *E. bromicola*-inoculated (KE+) barley plants to elucidate the association between *E. bromicola* colonization and barley grain size. Quantitative grain morphometric analysis further showed that grain width and grain area increased in KE+ by 6.8% and 5.2% compared with KE-, respectively. Structural analyze revealed that *E. bromicola* increased starch granule density in seeds. Phytohormone analyze showed that IAA enhanced nutrient transport by activating endosperm transfer cells, while ABA increased starch and storage protein synthesis for better grain filling. Integrated transcriptomic, and metabolomic analyses revealed that *E. bromicola* activated multiple hormone pathways, and enhanced the antioxidant system to regulate grain size. These findings suggest that inoculation with *E. bromicola* may improve barley grain yield performance. This study provides mechanistic insights into *E. bromicola-*mediated regulation of major seed agronomic traits and proposes a promising strategy for optimizing plant architecture by *E. bromicola* inoculation in sustainable agriculture.

## Introduction

Barley (*Hordeum vulgare* L.) is among the earliest domesticated and utilized cereal and forage crops in the world. Yield enhancement continues to be a central objective in barley breeding. Barley grain yield is largely determined by quantitative traits, including effective panicle number, grain number per panicle, and grain weight. with the latter being strongly influenced by grain size (Xing and Zhang, 2010). Grain size is critical determinants of cereal yield as they directly affect assimilate accumulation and final yield outcomes (Long et al., 2024). Grain size is not a single trait but the integrated outcome of multiple components, including grain length, width, and their associated developmental programs, which are differentially regulated across distinct growth stages (Gao et al., 2023). During grain development, the filling stage is especially critical, as it determines assimilate accumulation, endosperm development, and the final expression of grain morphology (Liu J. et al., 2022; Ma et al., 2023; Shen et al., 2023). Therefore, understanding how filling-stage regulatory networks shape grain morphology, particularly traits related to grain width and grain weight, is essential for deciphering yield-related variation in cereals (Zhang et al., 2020).

Endophytic fungi of the genus *Epichloë* are widespread inhabitants of internal plant tissues and can influence host growth, stress tolerance, immune responses, metabolic reprogramming, and phytohormone homeostasis (Kuźniar et al., 2025; Watts et al., 2023). In grasses, *Epichloë* endophytes are of particular interest because they establish persistent, often vertically transmitted associations that can substantially influence host physiology and adaptive capacity throughout development (Desai et al., 2025; Liu B. et al., 2022). Although *Epichloë* spp. are not naturally present in domesticated barley (Yi et al., 2018), they have been identified in wild barley (*Hordeum brevisubulatum*) and can be experimentally inoculated into barley (*Hordeum vulgare* L.) cultivars (Li et al., 2021; Wang et al., 2022). However, previous research has primarily focused on their effects on vegetative growth, abiotic stress tolerance, and defense-related responses, while their potential role in reproductive development, particularly grain formation, remains insufficiently characterized. The symbiotic association between barley and the seed - transmissible endophytic fungus *Epichloë bromicola* (Li et al., 2021; Malik et al., 2026) is a significant factor positively influencing the productivity and adaptability of barley to biotic and abiotic stresses (Wang et al., 2024; Liu et al., 2025). However, whether *E. bromicola* colonization directly influences barley grain morphology remains poorly understood (Chen et al., 2022).

Increased grain size contribute to the yield production (Guo et al., 2024). Grain filling is regulated not only by processes within the developing grain but also by assimilate transport and signaling interactions between maternal and filial tissues. Moreover, the spike, together with its associated vascular transport tissues, constitutes an important regulatory interface (Radchuk et al., 2023; Rutten et al., 2024). Phytohormone signaling, particularly auxin and its crosstalk with other hormones, plays central roles in endosperm development, seed morphogenesis, and filling-stage progression. In cereals, auxin-related signaling has also been implicated in endosperm differentiation and transfer-cell-associated regulation, and its spatial and temporal dynamics within the endosperm can directly influence grain shape, making it a plausible regulator of grain width (Hertig et al., 2020; Shirley et al., 2019; Zhang et al., 2020). At the same time, plant-microbe interactions are often accompanied by coordinated reprogramming of host hormone homeostasis, defense signaling, and metabolic pathways, suggesting that phytohormones may act as key intermediaries linking endophyte colonization to host developmental responses (Tripathi et al., 2025; Xu et al., 2018). Given that these processes operate across multiple biological layers, including physiological, transcriptional, and metabolic levels, single-level evidence is often insufficient to resolve their mode of action. Therefore, integrative approaches combining phytohormone profiling with multi-omics analyses are required to uncover the underlying mechanisms (Chen et al., 2022). To address these gaps, focusing on grain-filling spike tissue in conjunction with hormone, transcriptomic, and metabolomic analyses provides a robust framework for investigating how stable endophyte colonization may reshape regulatory networks associated with grain development.

Currently, there is a lack of systematic evidence demonstrating whether stable *Epichloë* colonization in barley progeny is associated with increased grain size and whether such effects are mediated through hormone-centered regulatory reprogramming in spike tissues during grain-filling. In this study, we used F_2_ barley progeny derived from coleoptile-inoculated plants and compared mature grain morphology in endophyte-infected (KE+) and endophyte-free (KE-) materials. Mechanistic analyses focused on spike tissues collected at 20 days after pollination. We combined grain phenotyping with phytohormone profiling, transcriptome sequencing, metabolome analysis, transcriptome-metabolome integration, and RT-qPCR validation to systematically assess endophyte-associated regulatory variation. We aimed to determine whether *Epichloë* endophyte colonization is associated with grain enlargement and, if so, to elucidate whether this phenotype is linked to hormone-centered transcriptional and metabolic remodeling in spike tissues during grain filling. Our findings will provide insights into the molecular mechanism of grain size regulated by *Epichloë* endophyte in barley.

## Materials and methods

### Strain and plant material

*E. bromicola* strain XJE1, originally isolated from wild barley, was provided by the Gansu Academy of Agricultural Science (Lanzhou, China). This strain is known to produce peramin, but not ergovaline, lolitrem B, and loline (Wang et al., 2025). Barley seeds (Kenpi 26) were sourced from the Gansu Academy of Agricultural Sciences. A novel *E. bromicola*-barley association was established following the protocols of Latch and Christensen (1985) and Li et al. (2021). Seeds collected from *E. bromicola-* inoculated plants were examined for *E. bromicola* infection through staining with aniline blue under the microscope (Saha et al., 1988; Liu et al., 2026). Following the method of Chen et al. (2019), endophytes were isolated from the seeds, and their morphological characteristics and DNA sequences were subsequently analyzed. Seeds collected from uninoculated (KE-) and inoculated (KE+) with *E. bromicola* plants were stored at 4 °C for future experiments.

### Field trials and sampling

Due to the limited number of seeds, F_1_ plants were initially cultivated under controlled greenhouse conditions (22/18°C day/night temperature, 10/14 h photoperiod, 800 μmol m^−2^ s^−1^photon flux density, and relative humidity of 65%). Plants were watered regularly and supplied weekly with half-strength Hoagland’s nutrient solution. At maturity, seeds were harvested from each plant, and endophytic fungi in the seeds were detected using staining and DNA-based methods as described above, respectively. F_2_ populations of *E. bromicola*- infected (KE+) and endophyte-free (KE-) barley were planted and phenotyped at the Huangyang Town Wheat Crop Experimental Station (N 37°40′1.33″, E 102°50′58.45″, altitude 1783 m), Gansu Province, China. The planting scheme was as follows: row spacing of 0.25 m, row length of 2 m, 5-row plots, 240 seeds per row, and three replicates. Prior to sampling, endophyte colonization status was verified microscopically in leaf sheath tissue stained with aniline blue (**Supplementary Figure 1**). Unless otherwise specified, spike tissues collected at 20 days after pollination (DAP) were used for phytohormone profiling, transcriptome sequencing, metabolome analysis, integrated multi-omics analysis, and RT-qPCR validation. Mature grains harvested at physiological maturity were used for grain morphology analysis. All experiments included at least three biological replicates, with each replicate consisting of pooled material from multiple plants.

### Grain morphology analysis

Mature grains from KE+ and KE- plants were collected at harvest. Grain images were acquired using an Epson Perfection V800 Photo flatbed scanner at 600 dpi. Grain length, grain width, and projected grain area were quantified using ImageJ (version 1.53k; National Institutes of Health, USA). At least 15 grains per treatment were analyzed across three biological replicates, each comprising grains collected from at least 5 plants. Additionally, agronomic traits including plant height, spike length, peduncle length, tiller number, spike number, and grain number per spike were recorded at physiological maturity. Abdominal endosperm cells of KE+ and KE- seeds were examined using conventional paraffin sectioning. Conventional paraffin section method was used to observe the changes in the internal structure of the seeds according to Al-Sabawy et al. (2021). Barley seeds were soaked in distilled water for 4 h, then transferred to FAA fixative solution (formalin: glacial acetic acid: 70% ethanol = 1:1:18) for 24 h. The seeds were dehydrated through a graded ethanol series and cleared using ethanol and xylene. The cleared materials were parrffin-embedded, double-stained with safranin and fast green, and then mounted with neutral balsam, placed in a 40 °C oven to dry. Each treatment was repeated five times, and five fields of view were selected per slide.

### Phytohormone quantification

Spike tissue collected at 20 DAP during the active grain-filling stage was immediately frozen in liquid nitrogen and stored at -80 °C. Approximately 0.3 g of fresh tissue was ground and extracted overnight with acetonitrile at 4 °C. Following centrifugation at 12,000 × g for 5 min, the supernatant was collected, and the residue was re-extracted twice. The combined extracts were purified using a C18 QuEChERS cartridge, evaporated to dryness under vacuum, reconstituted in 200 μL methanol, and filtered through a 0.22 μm membrane. Phytohormone analysis was performed on a Shimadzu LC-30AD HPLC system equipped with a Poroshell 120 SB-C18 column (2.1 × 150 mm, 2.7 μm) at 30 °C, coupled to an AB SCIEX Qtrap 6500 mass spectrometer. IAA, ABA, GA_3_, and trans-zeatin were quantified in multiple reaction monitoring (MRM) mode using authentic standards (Yuanye Bio-Technology, Shanghai, China) for calibration. Hormone concentrations were expressed as ng g^-^¹ fresh weight. Three biological replicates were analyzed per treatment.

### RNA extraction, library preparation, and transcriptome analysis

Total RNA was extracted from spike tissue at 20 DAP using TRIzol reagent. RNA quality and integrity were assessed using a NanoDrop 2000 spectrophotometer and an Agilent 2100 Bioanalyzer, and samples with RIN ≥ 7.0 were used for library construction. RNA-seq libraries were constructed and sequenced on an Illumina HiSeq platform to generate paired-end 150 bp reads. Raw reads were quality-filtered using Trimmomatic (version 0.36) to remove adapter sequences and bases with Q < 20. High-quality reads were mapped to the barley reference genome (*Hordeum vulgare* cv. Morex V3) using HISAT2 (version 2.1.0). Gene expression levels were quantified with StringTie (version 1.3.3b) and normalized as TPM (transcripts per million). Differential expression analysis was performed using DESeq2 (Love et al., 2014). For general transcriptome profiling, genes with FDR < 0.05 were considered significantly differentially expressed, whereas for downstream functional enrichment analyses, a more stringent threshold of FDR < 0.05 and |log_2_FC| ≥ 1 was applied. Principal component analysis was conducted using variance-stabilized transformed expressionvalues.

### GO and KEGG enrichment analysis

Gene Ontology enrichment analysis was performed using the top GO package in R, while KEGG pathway enrichment analysis was performed using cluster Profiler (Yu et al., 2012). GO terms and KEGG pathways with adjusted P values (Q values) < 0.05 after Benjamini–Hochberg correction were considered significantly enriched. Gene Ratio was calculated as the number of DEGs assigned to a term or pathway divided by the total number of DEGs in the corresponding analysis set. The most significantly enriched terms or pathways were visualized using bubble plots.

### Metabolite extraction, UHPLC-MS/MS analysis, and metabolomics data processing

Spike tissues at 20 DAP were vacuum-freeze-dried (Scientz-100F) and ground to powder using a mixer mill (MM 400, Retsch) at 30 Hz for 1.5 min. Approximately 50 mg of lyophilized powder was extracted with 1200 μL of pre-cooled (-20 °C) 70% aqueous methanol containing internal standards. Samples were vortexed for 30 s every 30 min (six times total), centrifuged at 12,000 rpm for 3 min, and the supernatant was filtered through a 0.22 μm membrane. UPLC-MS/MS analysis was performed on a Shimadzu LC-30A system coupled to an AB SCIEX TripleTOF 6600+ mass spectrometer. Chromatographic separation was achieved on a Waters ACQUITY Premier HSS T3 column (1.8 μm, 2.1 mm × 100 mm) at 40 °C using a gradient of 0.1% formic acid in water (A) and 0.1% formic acid in acetonitrile (B) at a flow rate of 0.4 mL/min. Data acquisition was conducted in information-dependent acquisition (IDA) mode under both positive and negative ionization conditions. Raw data were converted to mzXML format using ProteoWizard and processed with XCMS for peak detection, alignment, and retention time correction (Smith et al., 2006). Metabolite identification was performed using in-house and integrated public databases, as well as metDNA. Differential metabolites were identified based on variable importance in projection (VIP > 1) from OPLS-DA models and fold change (|log_2_FC| ≥ 1) (Galindo-Prieto et al., 2015; Trygg and Wold, 2002). Hierarchical clustering and Z-score normalization were applied for heatmap visualization.

### Integrated transcriptome-metabolome analysis

Integrated pathway-level analysis was conducted using Fisher’s combined probability test to assess concordance between transcriptomic and metabolomic KEGG results. For each pathway represented in both datasets, pathway-level P values derived from transcriptomic and metabolomic analyses were combined into a joint test statistic and were adjusted using the Benjamini–Hochberg method. Pathways with FDR < 0.05 were considered significantly enriched at the integrated level. Rich Factor was calculated as the ratio of enriched genes or metabolites to the total number of annotated features within a given pathway.

### RT-qPCR validation

RT-qPCR was performed to validate selected candidate genes identified through transcriptomic and multi-omics analyses. Total RNA from spike tissue at 20 DAP was reverse-transcribed using the PrimeScript RT Reagent Kit with gDNA Eraser. qPCR amplification was conducted on an ABI 7500 Real-Time PCR System using 2× SG Fast qPCR Master Mix (High Rox; BBI, Shanghai, China). Each 20 μL reaction contained 10 μL 2× premix, 0.8 μL each primer, 2 μL cDNA template, and 6.4 μL nuclease-free water. The amplification program consisted of an initial denaturation at 95 °C for 30 s, followed by 40 cycles of 95 °C for 5 s and 60 °C for 30 s, and a subsequent melting curve analysis. Relative gene expression was calculated using the 2^−ΔΔCt method with *HvActin* as the internal reference gene (Livak and Schmittgen, 2001). Three biological replicates and three technical replicates were assessed for each gene. Pearson correlation analysis was used to assess concordance between RNA-seq and qPCR-derived log_2_ fold changes.

Candidate genes were selected based on their functional relevance to the regulatory framework emerging from the multi-omics analyses, including genes associated with hormone signaling and host response (*DELLA*, LOC123420332; *TIFY6b*, LOC123396079; *WIR1A-like*, LOC123410024; *LURP15-like*, LOC123401509), and genes involved in metabolism/transport and redox support (*LSI2-like*, LOC123449706; *CAT1*, LOC123449777; *GDSL*, LOC123442517; *ATX1-like*, LOC123408376). Gene-specific primers were designed using Primer Premier 5.0, and sequences are provided in **Supplementary Table 1**.

### Statistical analysis

Unless otherwise specified, data are presented as mean ± SE. Differences between the KE+ and KE- groups were evaluated using two-tailed Student’s t tests. For omics-scale analyses and pathway-level integration, P values were adjusted using the Benjamini–Hochberg method to control the false discovery rate (Benjamini and Hochberg, 1995). All statistical analyses were performed in R (version 4.1.0), and significance thresholds were defined as *P < 0.05, P* < 0.01, and *P < 0.001.*

## Results

### Effect of *E. bromicola* on barley phenotypic characteristics

**Figure 1:**
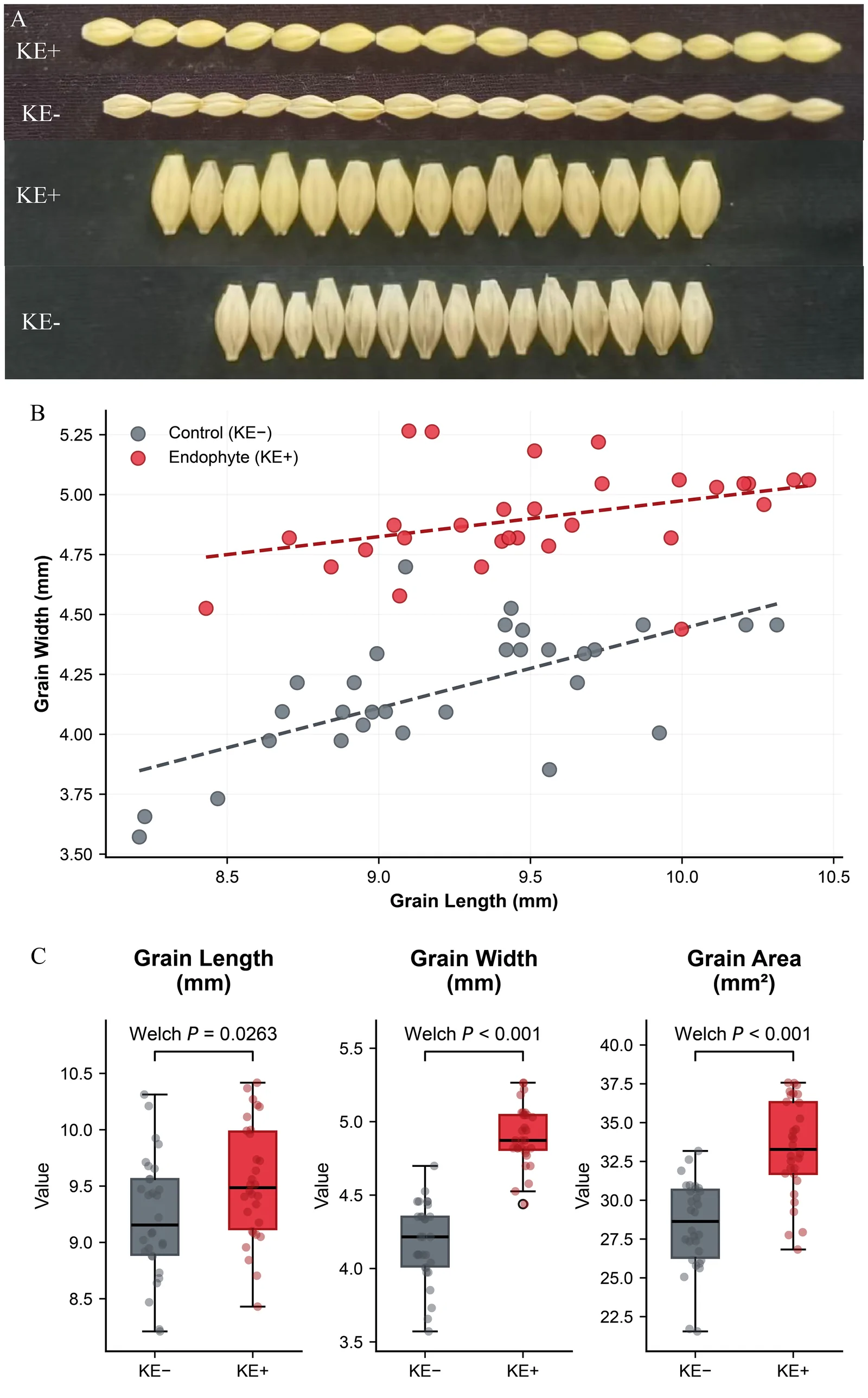
Effects of *E. bromicola* in F_2_ association barley plants. **(A)** Image showing differences between *E. bromicola* inoculated (KE+) and uninoculated barley plants (KE-) in grain shap. **(B)** Scatter plot showing the relationship between grain length and grain width between KE+ and KE-, Dashed lines indicate linear regression trends for each group. **(C)** Boxplots showing grain length, grain width, and grain area in KE− and KE+ grains. Statistical significance was assessed using Student’s t-test. *P < 0.05;* **P < 0.001.

**Figure 2:**
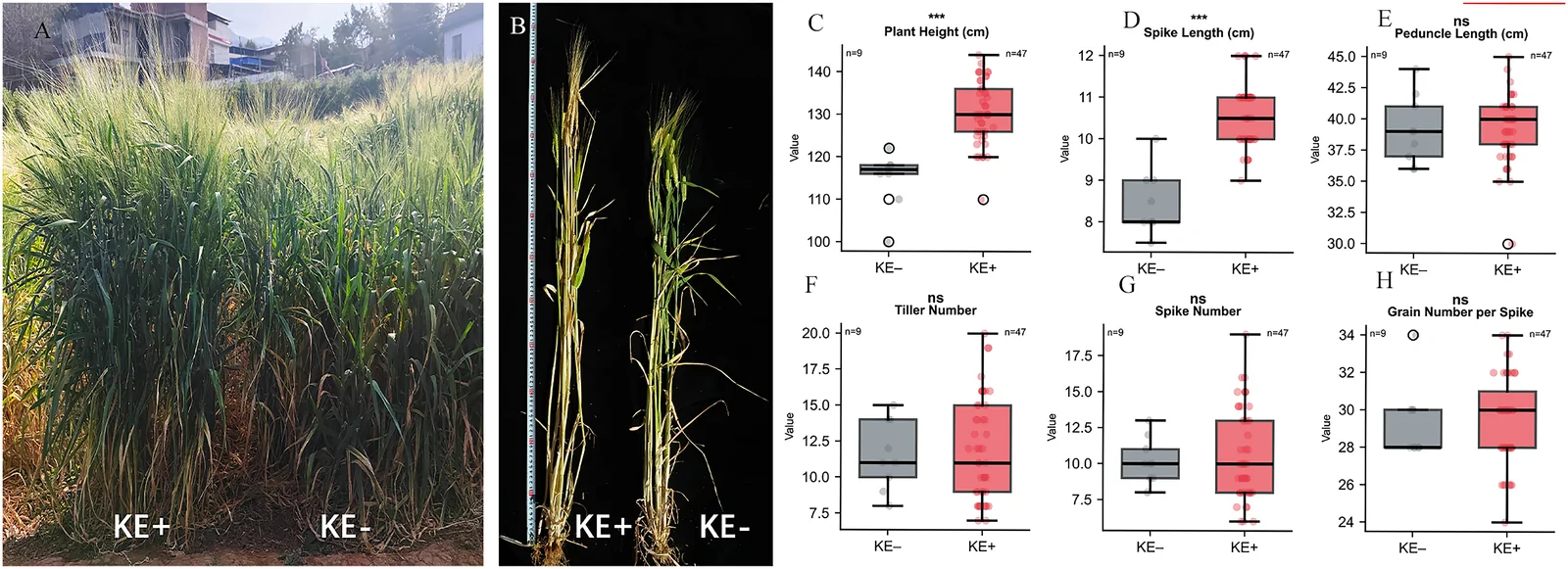
Endophyte treatment altered phenotype profiles in barley plants. **(A)** Assessment of *E. bromicola* in F_2_ inoculated (KE+) and uninoculated barley plants (KE-) in the field. Single-row field plots in Gansu, China. **(B)** Plant phenotypes of KE+ and KE- at grain-filling stage, significant differences were observed between *E. bromicola* inoculated (KE+) and uninoculated (KE-) plants in plant height **(C)**, spike length **(D)**, peduncle length **(E)**, tiller numbers per plant **(F)**, and **(G)** grain numbers per spike **(H)**. Statistical significance: Mann-Whitney U test (***p < 0.001, ns = not significant).

**Figure 3:**
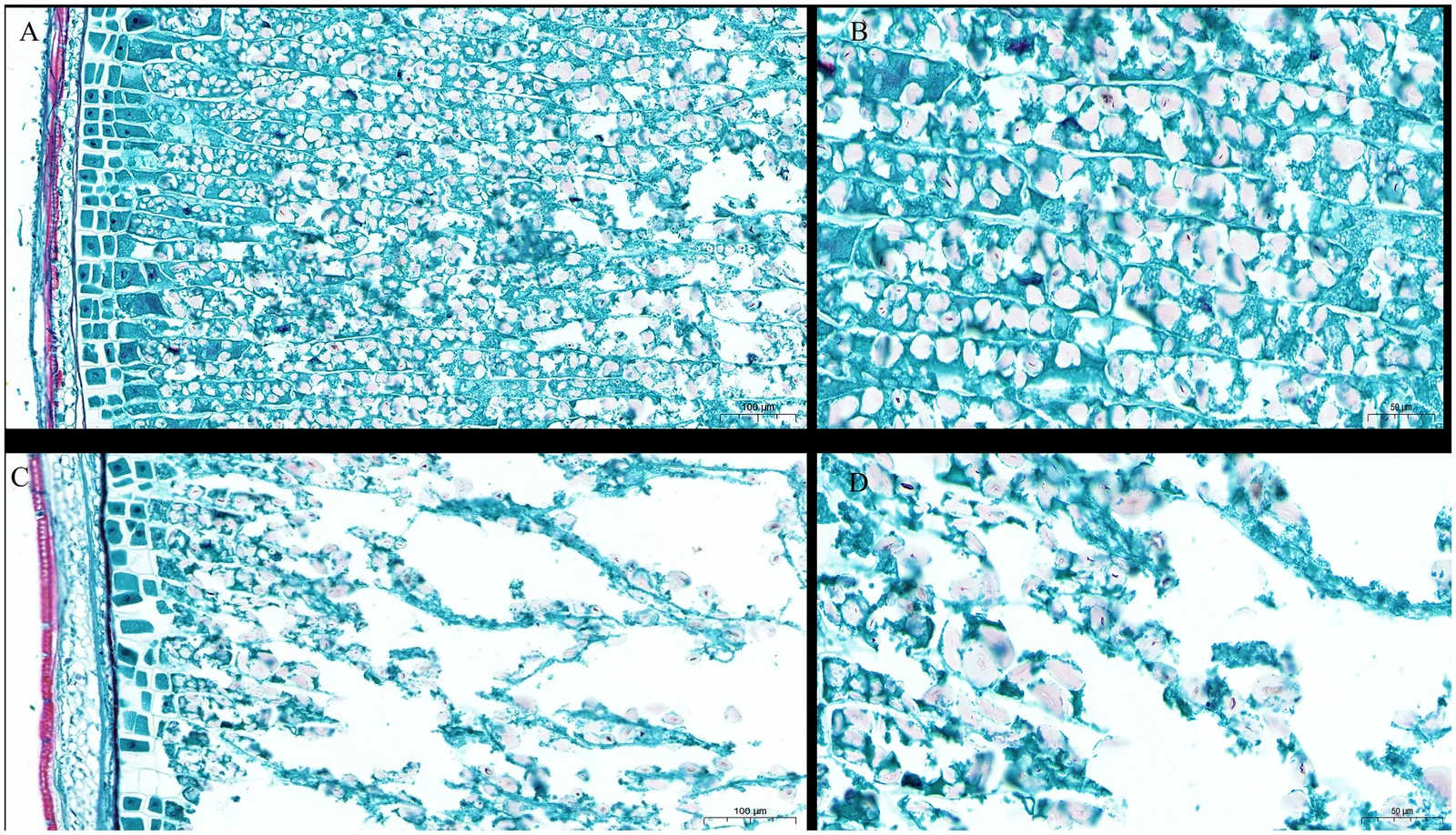
Endophyte treatment altered endosperm cells in barley seeds. **(A)** Abdominal endosperm cells of KE+ at maturity, Scale bars = 100 µm. **(B)** Enlarged endosperm cells in KE+, Scale bars = 50 µm. **(C)** Abdominal endosperm cells of KE- at Maturity, Scale bars = 100 µm. **(D)** Enlarged endosperm cells in KE+, Scale bars = 50 µm.

Compared with the corresponding non-colonized germplasm (KE-), the endophyte-inoculated barley line (KE+) produced visibly larger and fuller mature grains (**Figure 1A**). Notably, the grain length-width relationship displayed an upward shift in the KE+ group, indicating that grains of comparable length consistently exhibited greater width across the measured range (**Figure 1B**). Quantitative grain morphometric analysis further showed that grain width and grain area increased in KE+ by 6.8% and 5.2% compared with KE-, respectively (both with P < 0.001), whereas a relatively smaller increase of grain length, by 2.1% (P < 0.05), was observed (**Figure 1C**). Together, these results demonstrate that the enlarged-grain phenotype of KE+ was driven primarily by width-associated expansion rather than by proportional enlargement of all grain dimensions. This distinct morphological pattern establishes grain enlargement as a defining phenotypic feature of KE+ and provides the phenotypic foundation for subsequent mechanistic analyses. Beyond grain morphology, KE+ plants in the F_2_ generation also exhibited significantly increased plant height and spike length(**Figures 2A, B**), whereas peduncle length (**Figure 2E**), tiller number (**Figure 2F**), spike number (**Figure 2G**), and grain number per spike (**Figure 2H**) did not differ significantly from KE-. Interested, compared with KE- barley plant, KE+ plants significantly increased plant height (+10.3%, p < 0.001) and spike length (+15.7%, p < 0.001), with no significant changes in other traits (**Figures 2C, D**). In the KE+ seeds, the endosperm is rich in inclusions, which are evenly distributed throughout the endosperm (**Figures 3A, B**). In the KE- seeds, the endosperm cells become loosely arranged, with enlarged intercellular spaces, and the inclusions within the cells become smaller in size and fewer in number (**Figures 3C, D**).

### *E. bromicola* induced changes in barley phytohormones

**Figure 4:**
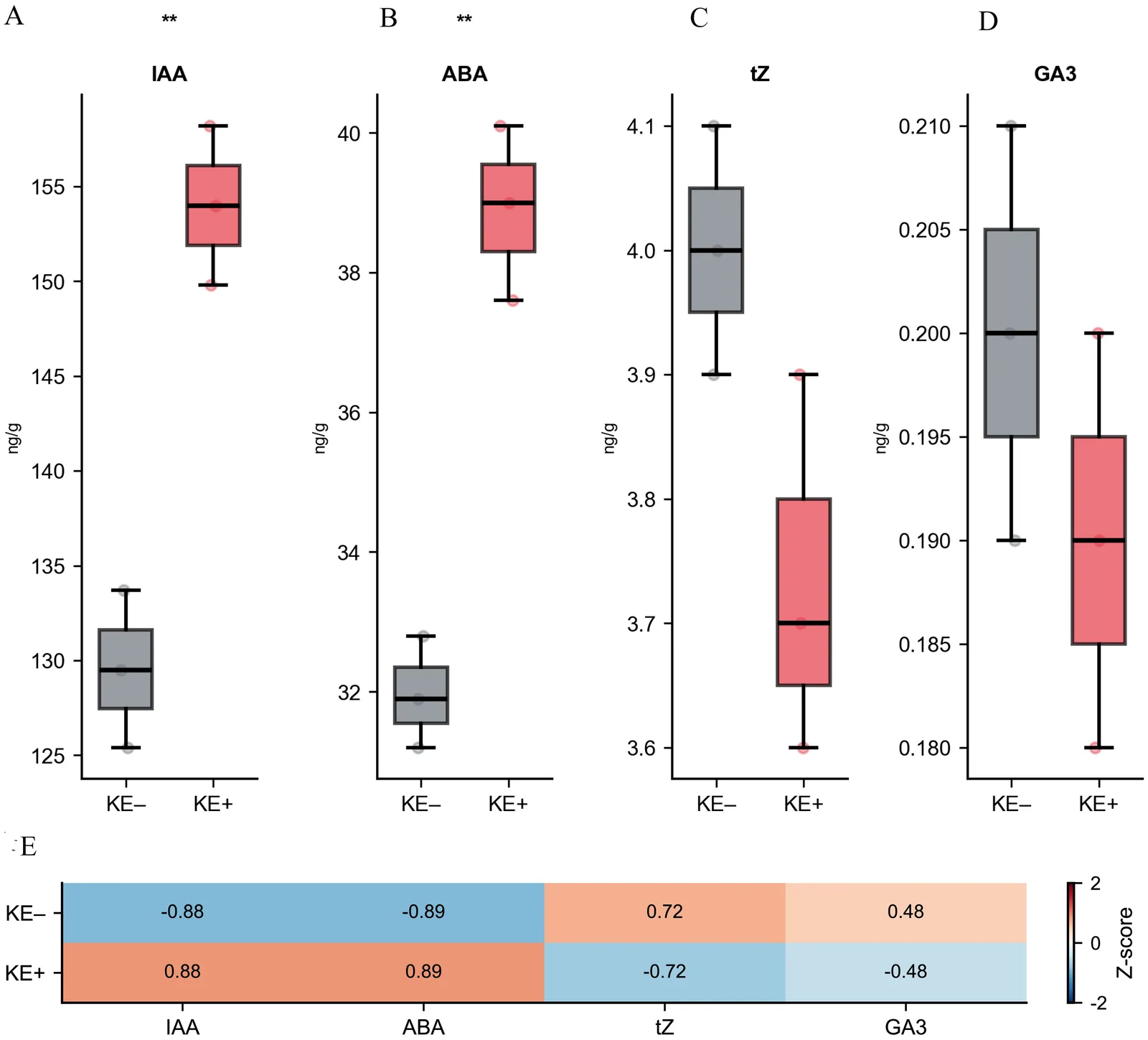
Endophyte treatment altered phytohormone profiles in rachis tissue. Boxplots of IAA **(A)**, ABA **(B)**, tZ **(C)**, and GA3 **(D)** contents in rachis tissue at 20 DAP from uninoculated (KE-) and *E. bromicola* inoculated (KE+) plants. Z-score heatmap showing the relative phytohormone profiles in the two groups **(E)**. Significance was determined by Student’s t-test (**P < 0.01).

To investigate whether the enlarged-grain phenotype observed in endophyte-colonized germplasm (KE+) was associated with altered hormonal regulation during grain filling, we quantified four major phytohormones (IAA, ABA, tZ, and GA3) in spike tissue at 20 days after pollination (DAP), a critical stage for grain filling. Compared with the corresponding non-colonized germplasm (KE-), KE+ spikes exhibited significantly higher IAA and ABA contents, with increases of 45.2% and 28.7%, respectively (**Figures 4A, B**). In contrast, tZ and GA3 levels showed no significant differences between the two groups (**Figures 4C, D**). Consistent with these individual comparisons, Z-score normalization of the hormone profiles further highlighted a pronounced shift between KE+ and KE-, with IAA and ABA showing positive Z-scores in KE+ (0.88 and 0.89, respectively) and corresponding negative Z-scores in KE- (-0.88 and -0.89) (**Figure 4E**). Notably, the magnitude of the IAA increase exceeded that of ABA, suggesting that auxin accumulation may represent the dominant hormonal shift associated with the KE+ phenotype. Collectively, these findings demonstrate that *E. bromicola* colonization was associated with selective remodeling of spike phytohormone homeostasis during grain filling, thereby providing a physiological basis for subsequent transcriptomic analysis.

### *E. bromicola* induces transcriptomic reprogramming in barley spikes

**Figure 5:**
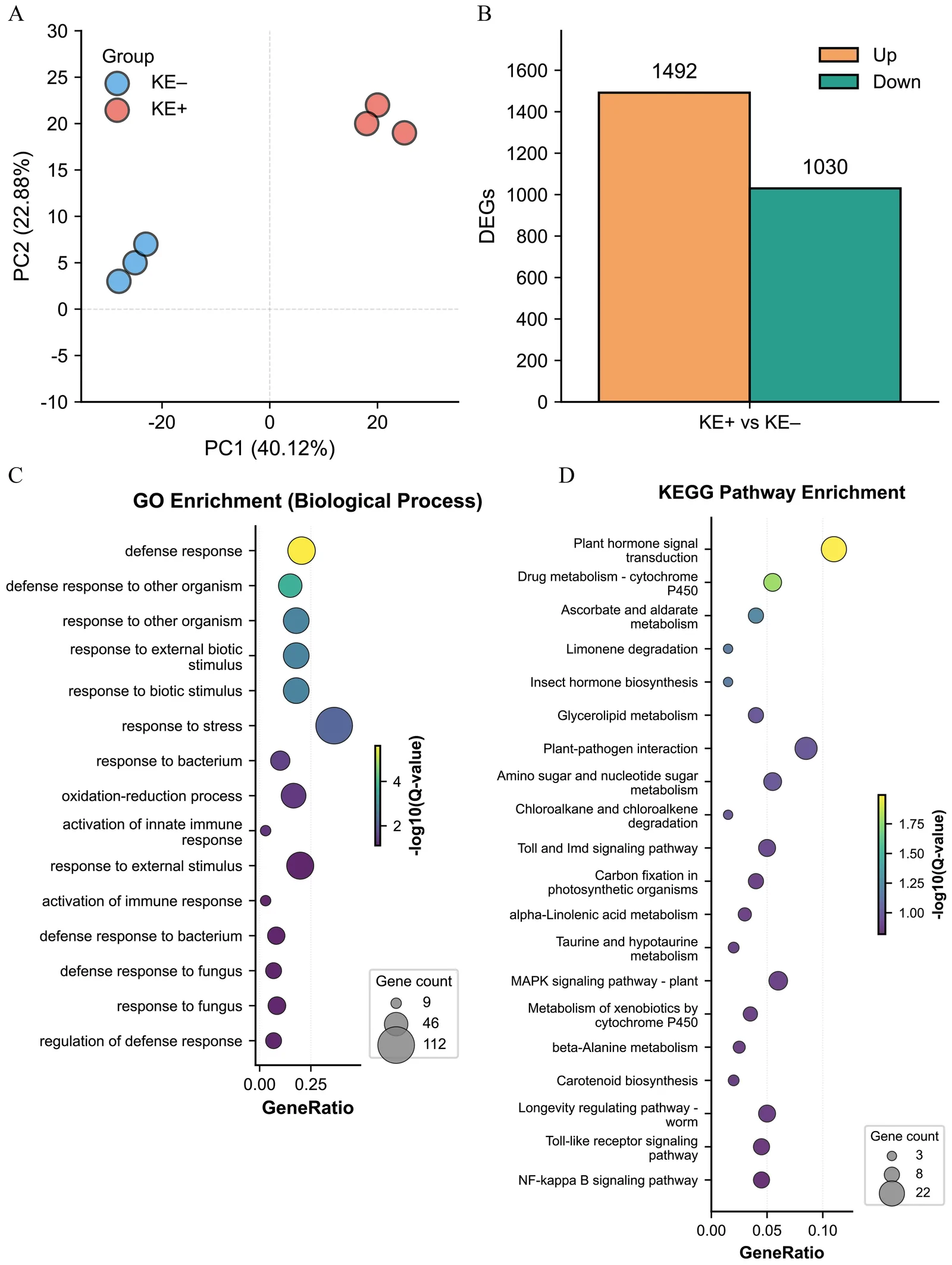
Transcriptome profiling and functional enrichment in KE- and KE+ spikes tissue. **(A)** PCA of transcriptome data from spikes tissue. **(B)** Number of DEGs identified between KE+ and KE−. **(C)** Top 15 enriched GO Biological Process terms among DEGs. **(D)** Top 20 enriched KEGG pathways among DEGs.

To determine whether the observed phytohormone alterations and enlarged-grain phenotype observed in the endophyte-colonized germplasm (KE+) were accompanied by broader molecular reprogramming during grain filling, we performed RNA-seq analysis on spike tissue of KE+ and non-colonized (KE-) plants at 20 DAP. Principal component analysis (PCA) revealed a clear separation between KE+ and KE- samples, with PC1 and PC2 explaining 40.12% and 22.88% of the total variance, respectively (**Figure 5A**). Together, PC1 and PC2 accounted for 63.00% of the total transcriptome variation, and biological replicates clustered tightly within groups, indicating high reproducibility and robustness of the transcriptome dataset. Differential expression analysis further identified 2,522 differentially expressed genes (DEGs; FDR < 0.05) between KE+ and KE-, including 1,492 upregulated and 1,030 downregulated genes in KE+ (**Figure 5B**). The predominance of upregulated genes (59.2%) suggests that endophyte colonization was primarily associated with transcriptional activation rather than repression in spike tissues.

GO and KEGG enrichment analyses were subsequently performed on the DEGs to elucidate the underlying biological processes and pathways. GO enrichment analysis showed that the DEGs were predominantly enriched in defense-, stress-, and immune-related biological processes (**Figure 5C**). Defense response emerged as the most significantly enriched GO term, followed by response to other organisms, response to stress, response to external biotic stimulus, and other immune-associated categories. These results indicate broad activation of biotic interaction- and stress-responsive transcriptional programs in KE+ spikes.

Supplementary GO analyses in the Molecular Function category showed significant enrichment of terms associated with oxidoreductase-related activities (45 genes; Q < 0.05) and transporter functions (**Supplementary Figure 2A**), while enriched Cellular Component terms were primarily associated with plasma membrane (75 genes) and cell periphery (87 genes) (**Supplementary Figure 2B**). These results suggest that the transcriptomic reprogramming in KE+ not only involved defense-related biological processes, but also was accompanied by changes in membrane-associated functions and transport- or redox-related activities.

KEGG pathway enrichment analysis identified plant hormone signal transduction as the most significantly enriched pathway (Q < 0.05; GeneRatio ≈ 0.10; 22 genes), followed by pathways involved in signaling and metabolic adjustment, including plant-pathogen interaction, MAPK signaling pathway, drug metabolism-cytochrome P450, and several primary-metabolism related pathways such as amino sugar and nucleotide sugar metabolism, glycerolipid metabolism, and alpha-linolenic acid metabolis. Plant hormone signal transduction was the most significantly enriched pathway (22 genes, Q = 0.0101) (**Figure 5D**). The prominent enrichment of plant hormone signal transduction pathways provides transcriptome-level support for the phytohormone shifts observed in KE+, particularly the marked increase in IAA detected in spike tissues (**Figure 4A**). In addition, the enrichment of multiple metabolism-related pathways suggests that hormone-associated transcriptional regulation was accompanied by broader metabolic reprogramming during grain filling. Together, these findings indicate that *E. bromicola* colonization elicits coordinated activation of defense-related processes and hormone-responsive signaling networks, establishing the molecular basis for subsequent metabolomic and integrative analyses.

### *E. bromicola* endophyte colonization alters barley metabolism

**Figure 6:**
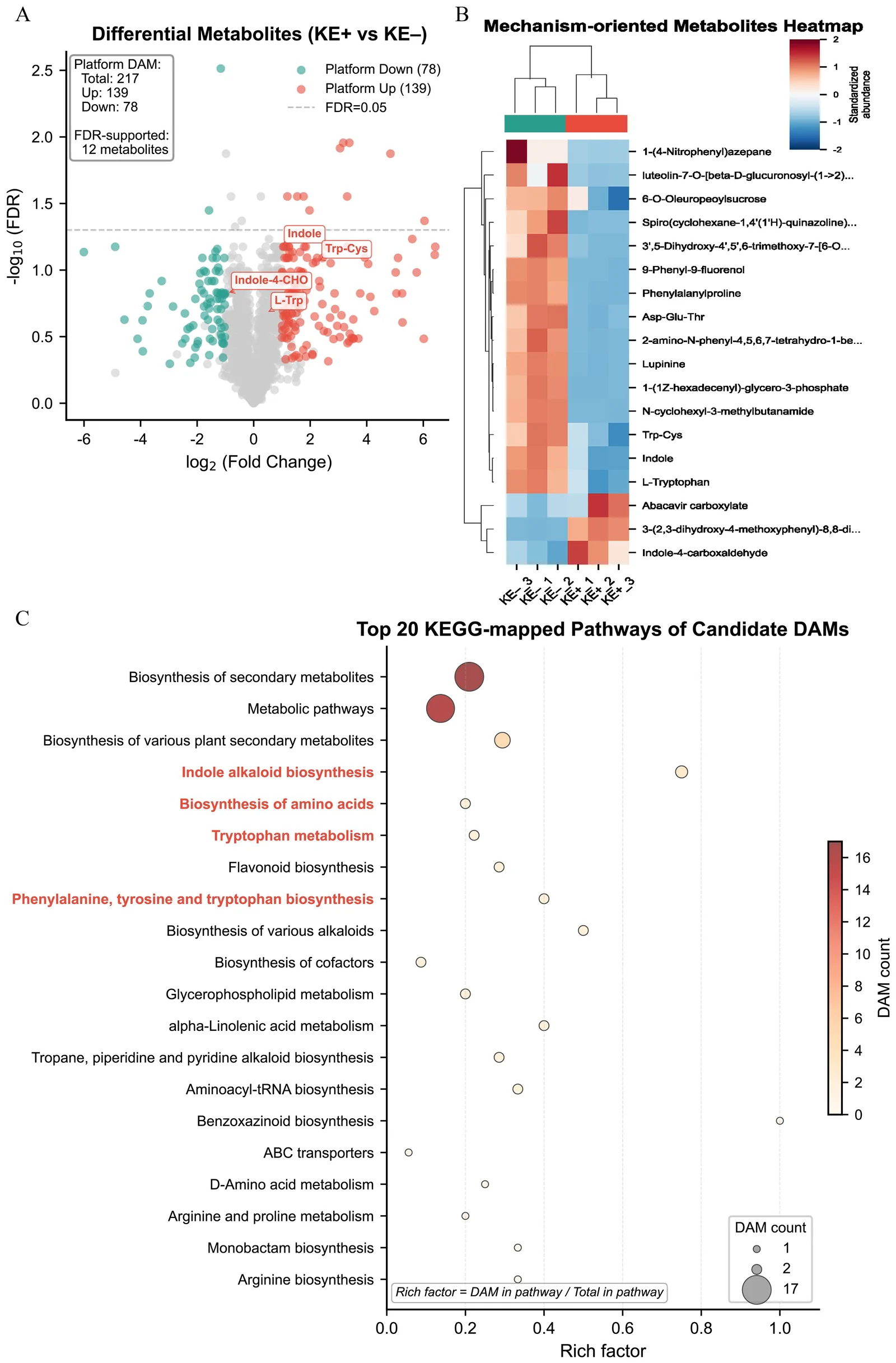
Metabolomic differences between KE+ and KE- and pathway-level representation of candidate DAMs. **(A)** Volcano plot of platform-defined differential metabolites between KE+ and KE-. Selected tryptophan/indole-related metabolites are labeled. **(B)** Heatmap of mechanism-oriented metabolites selected from the platform-defined DAMs. **(C)** Top 20 KEGG-mapped pathways of candidate DAMs. Red labels mark pathways of particular relevance to the tryptophan/IAA-related and secondary metabolism-related modules.

To examine whether the hormone-associated transcriptional reprogramming in endophyte-colonized germplasm was accompanied by corresponding metabolite changes, we conducted non-targeted UHPLC-MS/MS metabolomic profiling of spike tissues at 20 DAP. Differential metabolite analysis identified 217 significantly altered metabolites between KE+ and KE-, including 139 upregulated and 78 downregulated metabolites, indicating a predominant pattern of metabolite accumulation in KE+ (**Figure 6A**). These differential metabolites were initially classified according to the platform-assigned Type categories (sig/up/down), based on the combined criteria of VIP and fold change. Applying a more stringent multiple testing correction, 12 metabolites were significant at FDR < 0.05, suggesting that the overall directional shift was broader than the subset of strictly FDR-supported changes.

Notably, several metabolites associated with the tryptophan/indole module were prominently represented among the differential metabolites (**Figure 6B**). Among these, L-Tryptophan (fold change = 2.02, P = 0.007), Indole (fold change = 2.35, P = 0.004), and Trp-Cys (fold change = 4.58, P = 0.005) were significantly upregulated in KE+, whereas Indole-4-carboxaldehyde showed a modest decrease (fold change = 0.50, P = 0.023). Hierarchical clustering of these mechanism-oriented metabolites revealed clear separation between KE+ and KE- samples, with KE+ exhibiting coordinated accumulation of multiple tryptophan/indole-related compounds, whereas a smaller subset displayed the opposite trend (**Figure 6B**). This pattern indicates that endophyte colonization induces reproducible, module-level metabolic reprogramming in spike tissues rather than isolated metabolite changes.

At the pathway level, candidate differential metabolites mapped preferentially to tryptophan metabolism, indole alkaloid biosynthesis, biosynthesis of amino acids, phenylalanine, tyrosine, and tryptophan biosynthesis, and plant hormone signal transduction (**Figure 6C**). Although these mappings do not represent formal statistically significant enrichment, the recurrent involvement of tryptophan- and hormone-related pathways, together with the elevated IAA content observed in KE+ (**Figure 4A**) and the transcriptomic enrichment of hormone-responsive pathways (**Figure 5D**), supports the hypothesis that *E. bromicola* colonization was associated with coordinated biochemical remodeling centered on an auxin-related regulatory framework. Collectively, these metabolomic results provide metabolite-level support for the proposed mechanism that tryptophan/indole-associated metabolic reprogramming contributes to IAA accumulation and, ultimately, to grain enlargement in endophyte-colonized barley.

### Integrated transcriptomic-metabolomic analysis reveals convergent regulation mediated by endophyte colonization

To evaluate the convergence between the transcriptome and metabolome at the pathway level, we performed integrated multi-omics analysis using Fisher’s combined probability test. This method combines pathway-level P-values from both datasets into a joint significance statistic, followed by Benjamini-Hochberg FDR correction.

**Figure 7:**
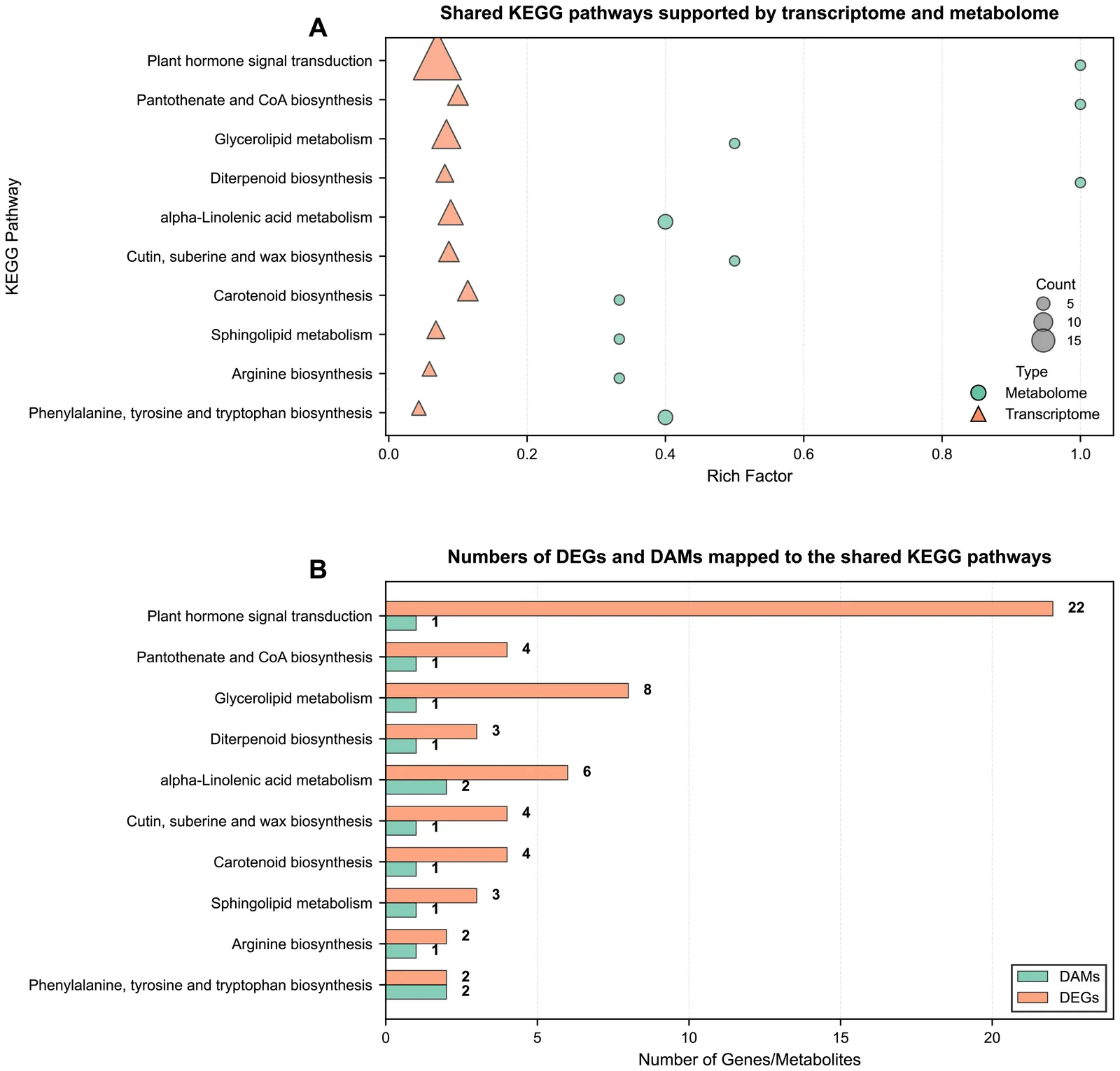
Integrated transcriptome-metabolome analysis identifies plant hormone signal transduction as the jointly significant pathway. **(A)** Bubble plot showing the top 10 shared KEGG pathways represented in both transcriptome and metabolome datasets. **(B)** Bar plot showing the numbers of DEGs (orange) and DAMs (teal) mapped to the same 10 shared KEGG pathways.

Among the 25 KEGG pathways represented in both -omics datasets, plant hormone signal transduction was the only pathway that retained joint statistical significance after multiple-testing correction (Fisher combined P = 1.32 × 10^-4^, FDR = 0.003) (**Figure 7A**). This pathway showed strong representation at the transcriptomic level (22 DEGs; P = 7.04 × 10^-5^) and modest representation at the metabolomic level (one differential metabolite; P = 0.150), with the combined evidence exceeding the significance threshold after FDR correction. Plant hormone signal transduction was the only pathway achieving joint significance (Fisher combined P = 1.32 × 10^-4^, FDR = 0.003), supported by 22 DEGs and 1 DAM. Two additional pathways, glycerolipid metabolism (FDR = 0.114) and alpha-linolenic acid metabolism (FDR = 0.114), exhibited suggestive joint enrichment but did not reach the conventional significance threshold (**Figure 7B**).

Notably, tryptophan metabolism, despite its biological relevance to IAA biosynthesis, did not achieve joint significance (Fisher’s combined *p* = 0.209, FDR = 0.476). This result reflects the relatively modest pathway-level P-values observed in both transcriptome (*p* = 0.132) and metabolome (*p* = 0.404) analyses. However, individual metabolite-level changes within the tryptophan/indole module remained evident (**Figure 6A**), even though pathway-level joint significance was not obtained.

Taken together, these integrative results identify plant hormone signal transduction as the central pathway showing statistically supported convergence between the transcriptomic and metabolomic datasets. This convergence aligns with the observed phytohormone alterations in KE+ and further supports the involvement of hormone-responsive regulatory networks in the endophyte-associated enlarged-grain phenotype.

### Validation of key candidate gene expression by qPCR

**Figure 8:**
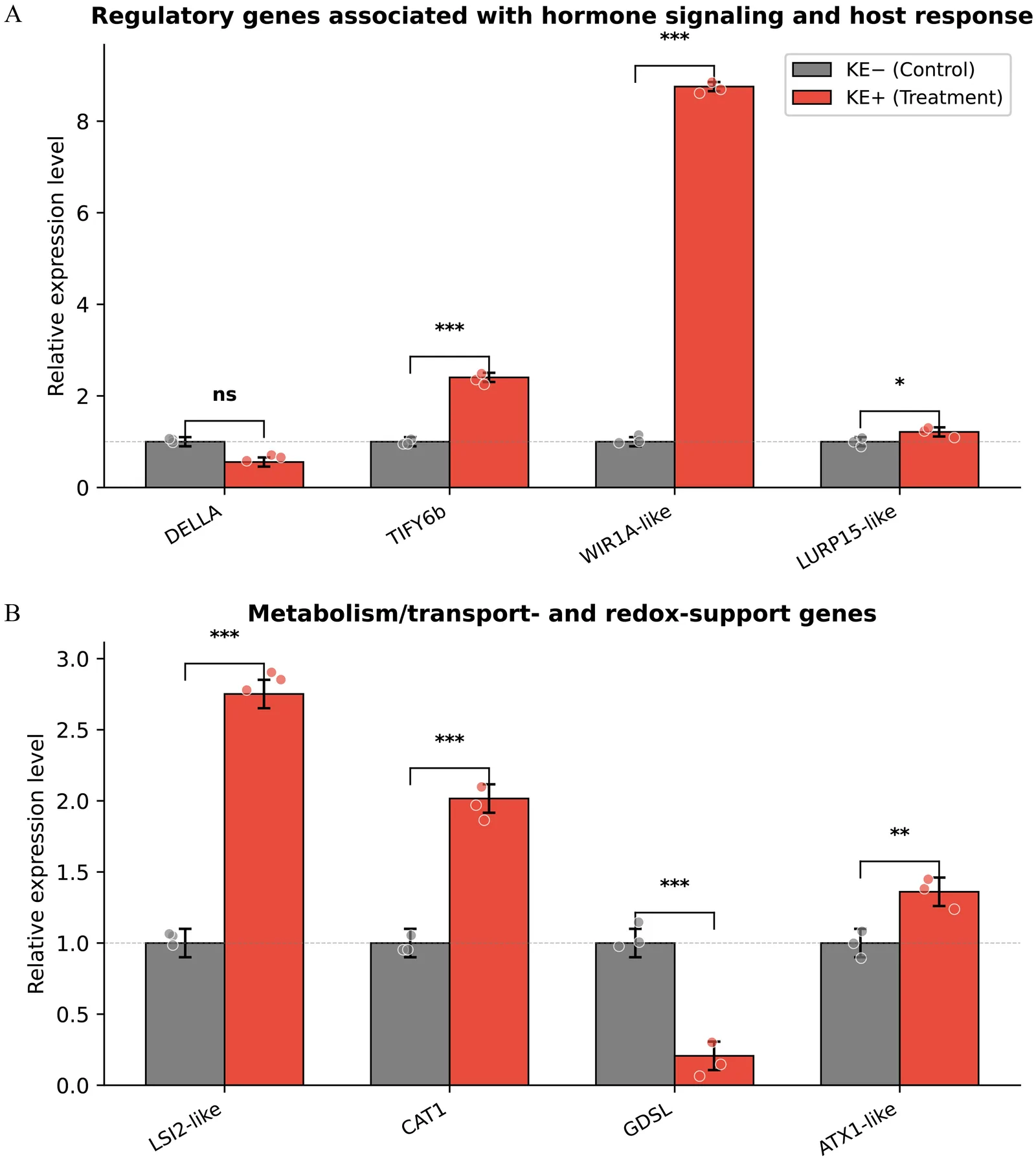
qPCR validation of selected candidate genes in spikes tissue. **(A)** Relative transcript levels of regulatory genes associated with hormone signaling and host response, including *DELLA*, *TIFY6b*, *WIR1A-like*, and *LURP15-like*. **(B)** Relative transcript levels of candidate genes related to metabolism/transport and redox support, including *LSI2-like*, *CAT1*, *GDSL*, and *ATX1-like*. Statistical significance between KE- and KE+ was determined using Student’s t-test: ns, not significant; *P < 0.05; P < 0.01; P* < 0.001.

To further validate the transcriptomic patterns identified by RNA-seq, we performed RT-qPCR analysis on eight selected candidate genes in spike tissues at 20 DAP. These genes were chosen from the DEG dataset based on their functional relevance to the regulatory framework that emerged from the multi-omics analyses, and were grouped into two categories: regulatory genes involved in hormone signaling and host responses (*DELLA*, *TIFY6b*, *WIR1A-like*, and *LURP15-like*), and genes related to metabolism/transport and redox support (*LSI2-like*, *CAT1*, *GDSL*, and *ATX1-like*) (**Figures 8A, B****).**

Among the regulatory genes, *WIR1A-like* exhibited the strongest induction in KE+ spike tissue, with an approximately 8.7-fold increase relative to KE- (*P <* 0.001*)*, indicating robust activation of host defense-related transcription in the endophyte-colonized germplasm. *TIFY6b* was also significantly upregulated (approximately 2.4-fold; *P* < 0.001), while *LURP15-like* exhibited a smaller yet statistically significant increase (*P < 0.05*) (**Figure 8A**). In contrast*, DELLA* displayed a downward trend that did not reach statistical significance, suggesting that not all hormone signaling-related candidate genes responded uniformly.

A distinct but coordinated pattern was observed for the metabolism/transport- and redox-related genes. *LSI2-like* and *CAT1* were significantly upregulated in KE+ (approximately 2.8-fold and 2.0-fold, respectively; both *P < 0.001)*, and*ATX1-like* also showed significant upregulation (*P* < 0.01). Conversely, *GDSL* was significantly downregulated (approximately 5-fold decrease; *P* < 0.001) (**Figure 8B**), indicating that endophyte colonization was associated with coordinated, yet directionally differentiated transcriptional adjustments in transport-, redox-, and metabolism-related functions.

To further assess the concordance between RNA-seq and qPCR, we compared the log_2_ fold changes for the eight candidate genes obtained from both methods (**Supplementary Figure 3A**). All genes displayed concordant directions of regulation, although the magnitude of change varied. In line with the qPCR results, *WIR1A-like* displayed the largest positive shift, whereas *GDSL* was the only gene consistently downregulated across both datasets. Correlation analysis revealed a strong positive relationship between RNA-seq-derived and qPCR-derived log_2_ fold changes (Pearson r = 0.914, P = 0.001, n = 8), with data points distributed closely along the 1:1 reference line (**Supplementary Figure 3B**). Collectively, these results provide independent gene-level validation of the RNA-seq results and reinforce the multi-omics inference that endophyte colonization is associated with coordinated regulatory and metabolic reprogramming in barley spike tissue.

**Figure 9:**
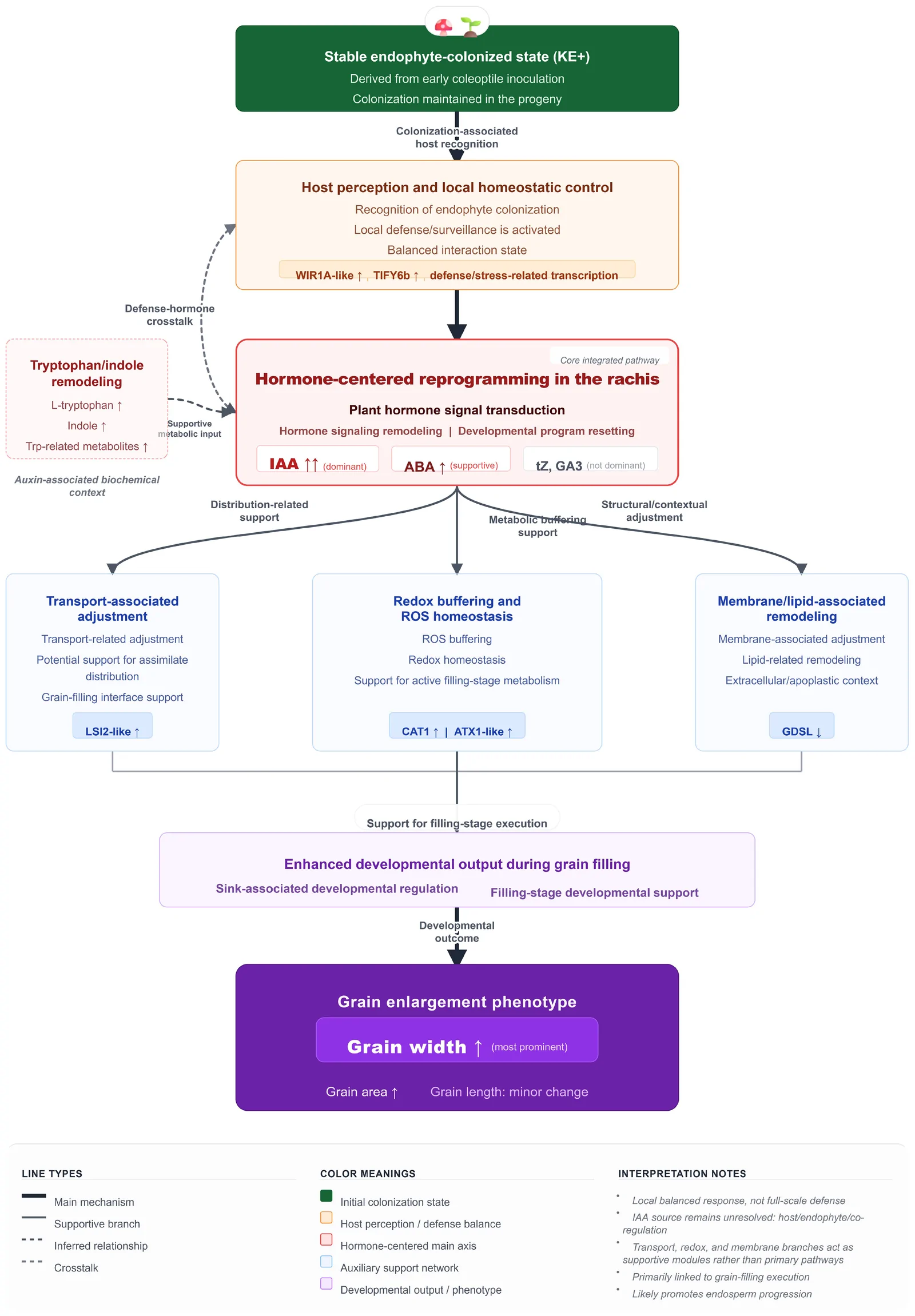
Mechanism of grain enlargement in barley by *E. bromicola* under field. The growth-promoting mechanisms of *E. bromicola* in barley involve IAA and ABA levels were increased, transport-associated adjustment (*LSI2-like*), redox buffering and ROS homeostasis (*CAT1*, *ATX1-like*), and membrane/lipid-associated remodeling (*GDSL*), which together are inferred to support filling-stage execution and sink-associated developmental regulation. Thick solid arrows indicate the main mechanistic chain, thin solid arrows indicate supportive branches, dashed arrows indicate inferred relationships or supportive inputs, and bidirectional dashed arrows indicate crosstalk. Upward (↑) and downward (↓) arrows indicate relative upregulation and downregulation, respectively, in KE+ compared with KE-.

Tryptophan/indole-related metabolic shifts might have created a supportive biochemical environment, the integrated analysis pinpointed hormone signaling as the most reliable mechanistic pathway. Specifically, increased IAA content enhances the activity of endosperm transfer cells, thereby improving nutrient transport efficiency. Concurrently, increased ABA content promotes the synthesis of starch and storage proteins, leading to better grain filling. In addition, higher CAT1 activity protected the integrity of aleurone layer cells, maintaining their capacity to produce hydrolytic enzymes essential for subsequent germination. Meanwhile downregulated GDSL expression reduces lipid breakdown, resulting in increased lipid storage. Collectively, these changes facilitated full expansion of endosperm cells and substantial accumulation of storage reserves, ultimately producing larger seeds with increased grain weight (**Figure 9**).

## Discussion

Overall, our results support a hormone-centered regulatory framework underlying endophyte-associated grain enlargement in barley, with the most pronounced morphological shift occurring in grain width. In KE+ plants, this width-biased phenotype was accompanied by selective phytohormone remodeling in spike tissue at 20 DAP, characterized by a marked increase in IAA and a moderate elevation in ABA, whereas tZ and GA3 remained largely unchanged. At the molecular level, both transcriptomic and metabolomic analyses revealed coordinated regulatory reprogramming involving hormone-related and tryptophan/indole-associated signals. Importantly, integrated pathway analysis revealed that plant hormone signal transduction was the sole pathway significantly represented in both transcriptome and metabolome datasets. This finding suggests that hormone-centered host remodeling constitutes the most robustly supported mechanistic axis underlying the KE+ phenotype. Because grain size in cereals is shaped by coordinated developmental patterning and filling-stage regulation, the present pattern places the KE+ phenotype within a biologically meaningful grain-development framework (Gasparis and Miłoszewski, 2023). Taken together, these findings support a hormone-centered model for endophyte-associated grain enlargement rather than a single-pathway explanation based on any one omics layer in isolation.

The preferential increase in grain width suggests that *E. bromicola* colonization primarily influenced filling-stage processes during grain development rather than early-stage developmental determinants of grain length. In cereals, grain size is established through multiple developmental windows, but final grain width and weight are strongly influenced by grain-filling dynamics, assimilate deposition, and hormone-regulated sink activity (Gasparis and Miłoszewski, 2023; Ma et al., 2017). The spike functions as a major transport and distribution hub within the cereal spike, featuring anatomically and functionally differentiated vascular routes that supply individual spikelets. Consequently, changes in spike physiology are likely to influence assimilate movement and final grain development (Radchuk et al., 2023; Rutten et al., 2024). In the present study, all hormone and multi-omics measurements were performed on spike tissue at 20 DAP, corresponding to the active grain-filling stage, thereby reinforcing the role of the spike as a key regulatory interface. The substantial accumulation of IAA in the KE+ spikes is therefore consistent with enhanced sink-associated developmental regulation during filling. In cereals, final grain width can reflect cellular processes operating during filling, including cell expansion, endosperm growth, and the coordinated development of maternal and filial tissues. Auxin is well known to regulate reproductive tissue growth, endosperm developmental progression, and seed morphogenesis, supporting the hypothesis that elevated IAA contributed to the width-biased grain phenotype through downstream effects on these processes (Shirley et al., 2019; Tuan et al., 2023). Notably, auxin signaling has also been implicated in endosperm developmental timing and filling-stage progression in cereals (Ma et al., 2017), providing a reasonable framework for the width-biased grain phenotype observed in this study. However, because the current dataset does not directly quantify assimilate flux, vascular unloading efficiency, or grain sink strength, these processes should be interpreted as plausible downstream consequences of spike hormone remodeling rather than experimentally demonstrated mechanisms.

Mechanistically, the integrated multi-omics dataset generated in our study positions hormone signaling at the center of the KE+ regulatory framework. In contrast, the concurrent remodeling of tryptophan/indole-associated metabolites provides a supportive biochemical context rather than an equally weighted alternative pathway. Among the quantified phytohormones, IAA showed the largest increase in KE+ spike tissues, consistent with the well-established role of auxin in ovule development, seed formation, and cereal grain growth (Hertig et al., 2020; Zhang et al., 2020). This interpretation is further supported by the recurrent identification of hormone-related signals across data layers. Notably, the plant hormone signal transduction pathway was significantly enriched in the transcriptomic dataset and remained the only pathway retaining significance following integrated transcriptome-metabolome analysis. Concurrently, KE+ spike tissues exhibited coordinated accumulation of L-tryptophan, indole, and related metabolites, indicating that endophyte colonization was associated with reproducible remodeling of a tryptophan/indole metabolic module. This metabolic shift is biologically relevant, as auxin biosynthesis and homeostasis are tightly connected to tryptophan-dependent and indole-containing intermediates in both plants and microorganisms (Gomes and Scortecci, 2021; Rico‐Jiménez et al., 2023; Tang et al., 2023). In addition, previous studies have demonstrated that beneficial plant-associated microorganisms can synthesize auxin or auxin-related indole compounds, thereby modulating host auxin signaling or compatibility states (Chen et al., 2023; Hilbert et al., 2012; Keswani et al., 2020). Nevertheless, because tryptophan metabolism itself did not remain significant in the integrated analysis, the most robust interpretation of our data is that tryptophan/indole remodeling functioned as a precursor-associated biochemical context that supports, but is subordinate to, the more strongly evidenced activation of hormone signaling pathways. Importantly, the lack of joint significance does not imply that non-overlapping pathways were biologically irrelevant; rather, some processes may have been preferentially captured in individual omics layers or fall below statistical thresholds in integrative analyses despite retaining functional importance. Accordingly, these metabolic changes may have contributed to IAA accumulation in KE+ spikes, but they should not be interpreted as definitive evidence for the primary source of auxin in this system.

A notable feature of the KE+ transcriptome was the strong representation of host-response and defense-related signals, including pronounced induction of *WIR1A-like* and enrichment of defense/stress-associated functional categories. At first glance, this pattern appears paradoxical as beneficial endophytes are often expected to suppress host defense responses. However, current models of beneficial plant-microbe interactions indicate that mutualists and endophytes are often initially perceived through host immune surveillance and can establish a dynamic equilibrium in which elements of defense signaling remain active without triggering detrimental outcomes (Desai et al., 2025; Oukala et al., 2021; Zamioudis and Pieterse, 2012). Within this framework, the KE+ phenotype may reflect a regulated coexistence state in which partial activation of host defense pathways occurs alongside, and potentially contributes to, developmental benefits during grain filling. Importantly, such a balance is likely to be spatially and temporally dynamic rather than static. This interpretation is also consistent with studies showing that beneficial endophytes can simultaneously modulate defense gene expression, antioxidant systems, and phytohormone profiles while maintaining or improving plant growth (Ahmad et al., 2022; Waqas et al., 2012). Moreover, concurrent modulation of IAA and ABA levels is consistent with the extensive crosstalk between growth- and defense- -related pathways, which are extensively integrated through phytohormone crosstalk rather than operating as strictly antagonistic systems (Tripathi et al., 2025; Xu et al., 2018). Because no pathogen challenge or resistance assays were conducted in the present study, this interaction should be described as a balanced or priming-like state rather than as direct evidence of enhanced disease resistance.

Beyond hormone signaling and host-response activation, KE+ spike tissues also exhibited transcriptional adjustments associated with transport processes, redox homeostasis, and membrane-related functions, suggesting that endophyte-associated grain enlargement was embedded within a broader physiological support network. In particular, genes such as *LSI2-like*, *CAT1*, and *ATX1-like* were upregulated, whereas *GDSL* was strongly downregulated, indicating coordinated but non-uniform remodeling of cellular support functions in KE+ spike tissues. This pattern is biologically plausible because active grain filling depends not only on developmental signaling, but also on efficient transport processes and sustained metabolic homeostasis (Ma et al., 2023; Radchuk et al., 2023). In rapidly developing or stress-adjusting plant tissues, elevated metabolic activity and defense-associated signaling are frequently accompanied by increased reactive oxygen species production, necessitating tight regulation to prevent oxidative damage while preserving redox signaling capacity (Ma et al., 2017; Wojtyla et al., 2016). Accordingly, the upregulation of *CAT1* in the KE+ spike is consistent with enhanced ROS-buffering capacity during active filling-stage adjustment. In parallel, changes in lipid- and membrane-associated functions are also plausible given their roles in maintaining membrane integrity, facilitating lipid turnover, and supporting extracellular or apoplastic remodeling during stress adaptation and developmental reprogramming (Jiang et al., 2023; Panozzo et al., 2025). Within this framework, the pronounced downregulation of *GDSL* may indicate a shift in lipid- or membrane-associated processes linked to cellular adjustment in KE+ spike tissues, although its precise functional significance remains to be elucidated. More broadly, studies in barley and other cereals show that stress-responsive and hormone-responsive tissues often exhibit overlapping changes in ROS detoxification, lipid metabolism, and transport-related pathways, supporting the biological plausibility of the observed transcriptional patterns (Berka et al., 2020; Svoboda et al., 2016). Collectively, these auxiliary networks likely facilitate physiological demands required for endophyte-associated grain enlargement. However, the candidate genes identified here should still be regarded as potential entry points for functional investigation rather than individually validated causal regulators of the grain phenotype.

From both agronomic and mechanistic perspectives, the present study establishes a link between a specific endophyte-host combination and a favorable reproductive phenotype in barley, characterized by increased grain size, particularly grain width. This finding is significant because grain size and grain filling are major determinants of cereal yield formation, and traits that improve sink development or filling efficiency may contribute to yield-related performance (Gasparis and Miłoszewski, 2023; Ma et al., 2023). A major strength of this work lies in the integration of multiple complementary datasets, including phenotypic measurements, phytohormone profiling, transcriptomics, metabolomics, integrated pathway analysis, and RT-qPCR validation. At the same time, several important limitations should be acknowledged. First, all molecular analyses were conducted in spike tissue rather than directly in developing grain tissues, leaving the precise spatial context of hormone-mediated regulation unresolved. Second, although IAA accumulation was clearly documented, the biological source of this auxin cannot yet be determined as host-derived, endophyte-derived, or jointly regulated. Third, while the multi-omics approach in this study provides strong correlative evidence, it does not establish direct causality between specific candidate pathways or genes and the grain enlargement phenotype. Future studies should therefore combine developmental time-course sampling with hormone quantification in grain tissues, histological analysis, isotope-assisted metabolic tracing, and targeted perturbation or functional validation of key candidate genes. More broadly, these findings align with the emerging concept that beneficial endophytes and microbiome-associated traits may provide useful biological leverage for crop improvement, although mechanistic validation remains essential before direct agronomic application can be claimed (Chen et al., 2022; Desai et al., 2025; Wolińska, 2025).

## Conclusion

Endophyte colonization was associated with a distinct enlarged-grain phenotype in barley, primarily characterized by increased grain width. Integrated evidence from phytohormone profiling, transcriptomics, metabolomics, and RT-qPCR validation supports a hormone-centered regulatory framework in spike tissues, with auxin-related processes emerging as the most prominent signal. Tryptophan/indole-associated metabolic changes likely provided a supportive biochemical context; however, hormone signaling remained the most robust mechanistic axis identified in the integrated analysis. These findings provide a conceptual framework for understanding endophyte-mediated regulation of grain development in barley, while highlighting the need for direct causal validation in future studies. In addition, further investigation is still needed on *E. bromicola*-barley association phenotypic and molecular characteristics in span of all growth stage.

## Data Availability

The raw RNA sequencing data have been deposited in the NCBI Sequence Read Archive (SRA). The names of the repository/repositories and accession number(s) can be found below: NCBI Sequence Read Archive (SRA), BioProject accession PRJNA1517570.
