# Supplementary material for: An Epichloë fungal endophyte regulates grain size in barley
Source: Front Plant Sci. 2026 Sep 10;17:1917832. doi: 10.3389/fpls.2026.1917832 (PMC13601360; doi:10.3389/fpls.2026.1917832)
Supplement: Supplementary file 1 [file DataSheet1.docx]

Supporting Information

Article title: **An Epichloë fungal endophyte helps plants to increase grain size through changs in hormone signalling**

Authors: Zhengfeng Wang^1*#^, Wanyou Liu^2, 3#^, Zhenjiang Chen^3^, Feng Zhao^4^,

Yonghong Qi^1^, Chunjie Li^3*^

The following Supporting Information is available for this article:

**Fig. S1.** Microscopic morphology of *E. bromicola* endophyte in barley leaf sheath.

**Fig. S2.** GO enrichment in the Molecular Function (A) and Cellular Component categories (B).

**Fig. S3.** Concordance between RNA-seq and qPCR expression changes for selected candidate genes.

**Fig. S1.** Microscopic morphology of *E. bromicola* endophyte in barley leaf sheath.

**Fig. S2.** GO enrichment in the Molecular Function (A) and Cellular Component categories (B).

Top 10 enriched GO terms of DEGs between KE and KE- in molecular function and cellular component.

**Fig. S3.** Concordance between RNA-seq and qPCR expression changes for selected candidate genes.

(A) Gene-wise comparison of log₂ fold changes (KE+/KE-) obtained from RNA-seq (blue) and qPCR (orange) for eight selected candidate genes. (B) Correlation analysis between RNA-seq and qPCR log₂ fold changes for the same genes.

**Table S1** Primer sequences used for RT-qPCR validation of candidate genes in barley spikes tissue, including the reference gene HvActin.

| Gene | Forward_primer | Reverse_primer | Forward_Tm | Reverse_Tm | Amplicon_bp |
| --- | --- | --- | --- | --- | --- |
| Actin | CGACAATGGAACCGGAATG | CCCTTGGCGCATCATCTC | | |  |
| LOC123396079 | ATCTTCTACGCTGGTTCTGTCAA | TGTGGCATCTGAGGTAGCATC | 58.6 | 58.3 | 203 |
| LOC123401509 | AATTCAAGACCGAGATGGATGT | CCAAGGTAGAAGACGCAGGA | 57.8 | 57.8 | 109 |
| LOC123408376 | CGTCCTCAAGGTGGCAATG | TTGCCCTTCACGGTCACC | 59.3 | 59.4 | 126 |
| LOC123410024 | ATCGCTCTCTTCGTCATCTCTG | ACCGACGCACACAGGACTAC | 58.7 | 58 | 111 |
| LOC123420332 | GCTGCGGTCTATCTCATCTGG | AGACACTAGTCGCGAGCATCAT | 59.8 | 59.2 | 118 |
| LOC123442517 | GGGCTGCGTTACCTGAATG | TCCACAAGCTCCCGTTCAC | 58.8 | 58.8 | 112 |
| LOC123449706 | GGAGAAGAGGTGGAAGGTGC | TGGGCGTCAGTGAAATCG | 58.4 | 58 | 150 |
| LOC123449777 | CCACCACAACAACCACTACGA | ACCTCTCCCCTGGCTGCT | 58.9 | 59 | 191 |
